# Plastic response of leaf traits to N deficiency in field-grown maize

**DOI:** 10.1093/aobpla/plac053

**Published:** 2022-10-27

**Authors:** Panpan Fan, Bo Ming, Niels P R Anten, Jochem B Evers, Yaoyao Li, Shaokun Li, Ruizhi Xie

**Affiliations:** Institute of Crop Science, Chinese Academy of Agricultural Sciences/Key Laboratory of Crop Physiology and Ecology Ministry of Agriculture, Beijing 100081, China; Center for Crop Systems Analysis (CSA), Wageningen University and Research, 6708PB Wageningen, The Netherlands; Institute of Crop Science, Chinese Academy of Agricultural Sciences/Key Laboratory of Crop Physiology and Ecology Ministry of Agriculture, Beijing 100081, China; Center for Crop Systems Analysis (CSA), Wageningen University and Research, 6708PB Wageningen, The Netherlands; Center for Crop Systems Analysis (CSA), Wageningen University and Research, 6708PB Wageningen, The Netherlands; Institute of Crop Science, Chinese Academy of Agricultural Sciences/Key Laboratory of Crop Physiology and Ecology Ministry of Agriculture, Beijing 100081, China; Institute of Crop Science, Chinese Academy of Agricultural Sciences/Key Laboratory of Crop Physiology and Ecology Ministry of Agriculture, Beijing 100081, China; Institute of Crop Science, Chinese Academy of Agricultural Sciences/Key Laboratory of Crop Physiology and Ecology Ministry of Agriculture, Beijing 100081, China

**Keywords:** Leaf area, leaf N content per unit leaf area, maize strategy, N deficiency, N management practices, specific leaf area

## Abstract

Nitrogen (N) utilization for crop production under N deficiency conditions is subject to a trade-off between maintaining specific leaf N content (SLN) important for radiation-use efficiency versus maintaining leaf area (LA) development, important for light capture. This paper aims to explore how maize deals with this trade-off through responses in SLN, LA and their underlying traits during the vegetative and reproductive growth stages. In a 10-year N fertilization trial in Jilin province, Northeast China, three N fertilizer levels have been maintained: N deficiency (N0), low N supply (N1) and high N supply (N2). We analysed data from years 8 and 10 of this experiment for two common hybrids. Under N deficiency, maize plants maintained LA and decreased SLN during vegetative stages, while both LA and SLN decreased comparably during reproductive stages. Canopy SLA (specific leaf area, cm^2^ g^–1^) decreased sharply during vegetative stages and slightly during reproductive stages, mainly because senesced leaves in the lower canopy had a higher SLA. In the vegetative stage, maize maintained LA at low N by maintaining leaf biomass (albeit hence having N content/mass) and slightly increasing SLA. These responses to N deficiency were stronger in maize hybrid XY335 than in ZD958. We conclude that the main strategy of maize to cope with low N is to maintain LA, mainly by increasing SLA throughout the plant but only during the vegetative growth phase.

## Teaser

How do maize plants adapt to external N deficiency conditions? The study was conducted in long-term N-fertilized field experiments and concluded that maize’s main strategy to cope with low N is to maintain leaf area, mainly by increasing specific leaf area (SLA) throughout the plant but only during the vegetative growth phase. The temporal and spatial distribution of SLA is also discussed in this study.

## Introduction

Nitrogen (N) deficiency is one of the most important abiotic factors reducing plant growth and crop yield because N plays a vital role in photosynthesis and crop productivity (Tollenaar and Lee 2002; Zhang *et al.* 2020). In agriculture, the amount and efficiency of use of N fertilizer in crops is an issue of intense scientific and societal debate. When the supply of N does not meet the potential demand of the crop, plants exhibit responses in leaf traits such as changes in leaf area (LA) production and senescence, specific leaf area (SLA; leaf area per mass) and N reallocation, which in turn influence crop growth and yield (Ellsworth and Reich 1993; Riha *et al.* 2013). Analysing these responses is important to elucidate the drivers of crop nitrogen-use efficiency.

Canopy-level response to reduced N uptake and implications for canopy photosynthesis can be viewed by considering that canopy photosynthesis is the product of light absorption and radiation-use efficiency (RUE, canopy photosynthesis/light capture) (Haxeltine and Prentice 1996; Bonelli and Andrade 2020). Plants can thus respond to low N uptake through maintenance in LA but reducing specific leaf N content per unit area (SLN). This results in light capture being maintained but a reduction in RUE, given the positive relationship between SLN and leaf photosynthesis (Field and Mooney 1986; Drouet and Bonhomme 2004). Conversely, SLN and RUE can be maintained, if LA and consequently in light capture are reduced (Grindlay 1997; Bonelli and Andrade 2020). An optimal balance in these two responses helps plants maximize photosynthesis for a given amount of canopy nitrogen (Anten *et al.* 1995). The strategies followed by plants to cope with N deficiency may differ both among and within plant species (Lemaire *et al.* 2008; Massignam *et al.* 2009). For instance, wheat, potato and canola have predominantly been found to reduce LA and associated light capture, thereby maintaining SLN and RUE (Vos and Van Der Putten 1998; Lemaire *et al.* 2008). By contrast, maize and tall fescue respond to N deficiency by a reduction in SLN (Vos *et al.* 2005; Massignam *et al.* 2011). Sunflower and sorghum have an intermediate response strategy, showing an almost equal decline in both LA and SLN (Lemaire *et al.* 2008; Oosterom *et al.* 2010). Although broad trade-offs among LA and SLN have been demonstrated, both LA and SLN typically decrease with crop development since leaf senesces and N reallocation to grain for protein synthesis during reproductive stages (Bertheloot *et al.* 2008; Li *et al.* 2022). We do not know how these responses are mediated by underlying plant traits and the extent to which they are dependent on the developmental stage of the plants. The extent of intraspecific variation in these responses is also not known.

Plants can alter their intraspecific morphological and physiological traits to acquire and use limited available resources through phenotypic plasticity in response to external environment change (Poorter *et al.* 2009; Legner *et al.* 2014; Zhu *et al*. 2015). An important leaf trait in this respect is SLA (cm^2^ g^–1^), as it reflects the trade-off between light capture (LA) and mass (and hence photosynthetic compounds) per unit area (Wilson *et al.* 1999; Liu *et al.* 2017). For instance, plants typically respond to drought conditions by decreasing SLA, keeping smaller but thicker leaves to decrease water loss (Pérez-Ramos *et al.* 2013). The acclimation to shade is characterized by increased SLA because higher SLA provides more LA for light harvesting (Evans and Poorter 2001). The interception and spatial distribution of light within the canopy thus induce SLA adjustments, which in turn change the light environment within plant canopies (Archontoulis *et al.* 2011; Yao *et al.* 2016; Liu *et al.* 2017). Furthermore, SLA influences the amount of nitrogen per unit area (SLN) and hence the SLN profile in the canopy and it affects the N allocation between leaf structural components and leaf photosynthesis (Wilson *et al.* 1999; Mu *et al.* 2016). Therefore, the plasticity in SLA is crucial in determining canopy photosynthetic capacity and the photosynthetic utilization efficiency of light and N (Reich *et al.* 1998; Yao *et al.* 2016). The dynamics in canopy SLA are also affected by leaf age and plant size (Evans and Poorter 2001; Poorter *et al.* 2009). In most herbaceous species, canopy SLA increases during leaf expansion, after which there is a decline presumably because of a build-up of cell wall material and chloroplasts (Simioni *et al.* 2004; Milla and Reich 2007). Hence, capturing temporal and spatial distribution in leaf SLA has been a long-standing goal of ecological research and is also a crucial part of advancing crop models (Archontoulis *et al.* 2011; Serbin *et al.* 2019).

Maize (*Zea mays*) is one of the most extensively cultivated cereal crops worldwide and plays an important role in ensuring food security. However, maize is very sensitive to N deficiency under field conditions (Massignam *et al.* 2009; Bonelli and Andrade 2020). Therefore, identifying how maize plants respond and adapt to N-limited conditions in terms of leaf traits is critical to enhancing crop productivity and resource-use efficiency. Plants respond to N fertilizer only when soils have low N availability. Long-term field experiments, in which fertilizer treatments are maintained for 5–10 years, are therefore most suitable to test the effect of N fertilization. The general objective of this study was to quantify maize response to N-deficient conditions at the leaf and canopy level and in relation to plant ontogeny. Specifically, we address the following questions: (i) what is the trade-off strategy between LA and SLN to adapt to N deficiency in the maize canopy, (ii) does this strategy differ during vegetative and reproductive stages? (iii) How does SLA respond to N deficiency in leaf and canopy scale? To this end, our study focused on the comparative analysis of leaf traits at the plant and canopy levels for vegetative and reproductive stages in a long-term N-fertilized maize field.

## Materials and Methods

### The site and experimental design

This study was conducted in a long-term N-fertilized (urea) field, in which the N fertilizer management practices had been maintained since 2009. Our data were collected in Years 8 (2016) and 10 (2018) of the experiment. The experiment was carried out at Gongzhuling Experimental Station of the Chinese Academy of Agricultural Sciences (43°53N, 124°81E), which is located in Jilin province, Northeast China. Two of the most widely grown maize hybrids in China, XY335 and ZD958, were used in this study. Individual plots were 45.5 m^2^ in size and comprised seven rows of 10 m in length separated by 0.65 m distance. The plant population density was 6.75 plants per m^2^. The plots were arranged in a randomized block design with three replications each. The seeds were sown on 29 April in both years. Weeds were controlled with herbicides. The experimental site received 602.3 and 635.2 mm of precipitation during the maize growing season (from 1 May to 30 September) in 2016 and 2018 and had a mean daily air temperature of 19.9 °C and 20.4 °C, respectively (Table 1).

**Table 1.** Mean temperature and precipitation during the growing season in the Years 2016 and 2018 at the experimental station.

Month	Year 2016		Year 2018	
	Temperature (°C)	Precipitation (mm)	Temperature (°C)	Precipitation (mm)
May	16.3 ± 4.3	173.0 ± 13.8	17.1 ± 3.5	87.1 ± 7.5
June	20.8 ± 2.7	102.2 ± 8.1	22.3 ± 2.7	121.9 ± 8.3
July	23.8 ± 2.1	62.5 ± 6.2	25.5 ± 2.5	128.1 ± 10.2
August	22.5 ± 3.9	132.5 ± 9.1	21.6 ± 2.5	246.7 ± 17.6
September	16.4 ± 3.4	132.1 ± 7.9	15.5 ± 3.7	51.4 ± 3.1

### N fertilizer treatments

The total N amount and application stage of the three N fertilizer management practices on the long-term N-fertilized field were as follows: no N fertilizer was applied throughout the whole growth period (N0), 150 kg N ha^−1^ was all applied as base fertilizer before sowing (N1), and 150 kg N ha^−1^ was applied as described for the N1 treatment, and an additional 150 kg N ha^−1^ split equally at V6 and silking stages (N2). P_2_O_5_ (super phosphate) 42.5 kg ha^−1^ and K_2_O (potassium sulfate) 42.5 kg ha^−1^ were applied as the base fertilizer for all treatments. The fertilizer was applied in the middle of two rows through traditional broadcasting. The topsoil (0–20 cm) at the experimental site was classified as chernozem, and the fundamental soil fertility of each growing season was measured before sowing (Table 2). Total nitrogen content was determined using an automatic meter (Kjeltec 8400, FOSS, Denmark) according to the Kjeldahl method (Bremner 1960).

**Table 2.** Total soil N content in the upper 0–20 cm before sowing.

Total N content	Year	N0	N1	N2
(g kg^−1^)	2016	1.08c	1.29b	1.46a
	2018	0.82c	1.11b	1.24a

Different letters in the same row indicate significant differences at *P*＜0.05 (*n* = 10). Nutrient availability of P and K were applicated the same for all N management treatments.

### Plant sampling and measurement

Plants in the three central rows (considering the border effect) were tagged. Leaf rank was counted from the bottom to the top, and tags were placed on leaves 4, 8 and 12 to avoid confusion as lower leaves were senesced (Fan *et al.* 2020). Focal plants were randomly selected among tagged plants and the whole plants were cut down at soil-surface level at the six-leaf stage (V6), 12-leaf stage (V12), silking stage (R1), milking stage (R3) and at physiological maturity (R6) (Broeske 2017). After cutting the fully expanded leaves from the tagged plants, the maximum length (*L*, cm) and maximum width (*W*, cm) of each leaf was measured with a ruler. The LA of every single fully expanded blade was calculated by Equation (1) with a coefficient of 0.75 (Zhen *et al.* 2018; Fan *et al.* 2020), and the LA at the plant level was calculated as the sum of every single fully expanded green leaf area.


$$\begin{array}{l} LA_{i}=0.75\times L\times W \end{array}$$ (1)

Individual leaves were dried at 85 °C to a stable weight, weighed and ground to a fine powder. Nitrogen concentration of each sample was determined using the Kjeldahl method (Bremner 1960).

### Calculations and statistical analysis

Canopy SLA was defined to be the ratio between green leaf area to the dry weight of leaf biomass (LB) (Equation 2), and canopy SLN was calculated as the amount of leaf N per unit of green leaf area (Equation 3).


$$\begin{array}{l} SLA=\underset{i=1}{\overset{n}{\sum }}\;LA_{i}/\underset{i=1}{\overset{n}{\sum }}\;LB_{i}\; \end{array}$$ (2)

$$\begin{array}{l} SLN=\underset{i=1}{\overset{n}{\sum }}\;TLN_{i}/\underset{i=1}{\overset{n}{\sum }}\;LA_{i}\; \end{array}$$ (3)

$$\begin{array}{l} TLN_{i}=LNC_{i}\times LB_{i} \end{array}$$ (4)

where LA is green leaf area (cm^2^), LB is leaf biomass (g), LNC is the leaf N concentration (mg g^–1^), TLN is the total leaf N amount (g), and in Equations (2) and (3), *n* is the total number of green leaves of the maize plant and the suffix *i* indicates an individual leaf value (e.g. LA_*i*_, LB_*i*_ is the leaf area and leaf biomass of the leaf rank *i*, while LA and LB are the leaf area and leaf biomass of the whole plant).

Specific leaf area (cm^2^ g^–1^, the leaf area per unit leaf dry biomass) is an essential indicator for estimating plant strategies in response to environmental changes (Legner *et al.* 2014; Zhou *et al.* 2020). Hence, the relationship between LA and LB at the whole plant level was examined by a power function in this study, as it shows how SLA scales with canopy size (Milla and Reich 2007).

A power function LB = *α* * LA^*β*^ (by ln-transforming form)


$$\begin{array}{l} \ln(LB)=\ln(\alpha )+\beta \ln(LA) \end{array}$$ (5)

*β* > 1 indicates that LB increases disproportionately with increasing LA and SLA decreases as LA increases; *β* < 1 means the opposite and *β* = 1 represents that SLA is unaffected by LA (Milla and Reich 2007; Zhou *et al.* 2020).

A three-way ANOVA test was used to evaluate the effect of N application (at levels N0, N1 and N2), hybrids (ZD958 and XY335) and growth stages (V6, V12, R1, R3 and R6) on leaf traits. A two-way ANCOVA test was used in Table 3 to analyse the effects of hybrids and N application on the relationship between LA and LB. The least significant difference was used to determine treatment differences at a *P* < 0.05 level of probability (R software, version 3.6.1). The ‘ggplot2’ package of R programming language was used to produce figures.

**Table 3.** Two-way ANCOVA (analysis of covariance) results in variance.

Effect	Year 2016				Year 2018			
	DFn	DFd	*F*-value	*P*-value	DFn	DFd	*F*-value	*P*-value
LA	1	83	217 766.8	3.14e-102*	1	83	14 537.95	5.46e-95*
Hybrids (H)	1	83	20.88	1.69e-05*	1	83	16.73	9.93e-05*
N application (N)	2	83	2.29	1.08e-01ns	2	83	0.03	9.72e-01ns
H × N	2	83	3.08	5.10e-02ns	2	83	0.005	9.95e-01ns

For both ZD958 and XY335, *β* values in Equation (5) were greater than 1 in both years, which suggested that LB tended to increase disproportionally faster than LA (Fig. 4). Consequently, canopy SLA decreased with increasing leaf size at the plant level.

## Results

### LA and canopy SLN variations under the N deficiency regime

**Figure 1.**
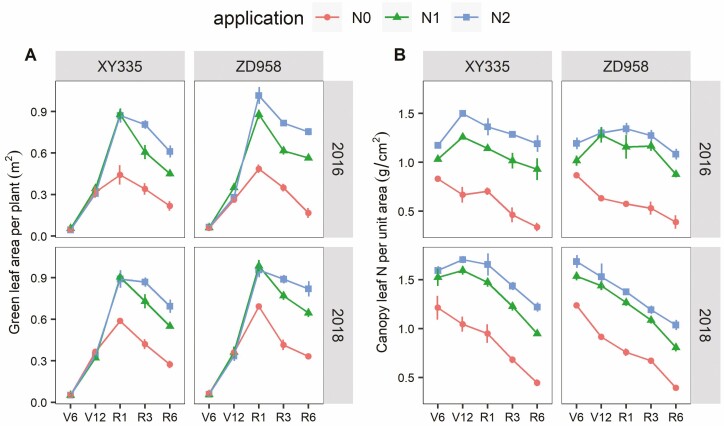
Green leaf area per plant (A) and the canopy leaf N per unit area SLN (B) at five growth stages among three N application rates of XY335 and ZD958 during the 2016 and 2018 growing seasons.

**Figure 2.**
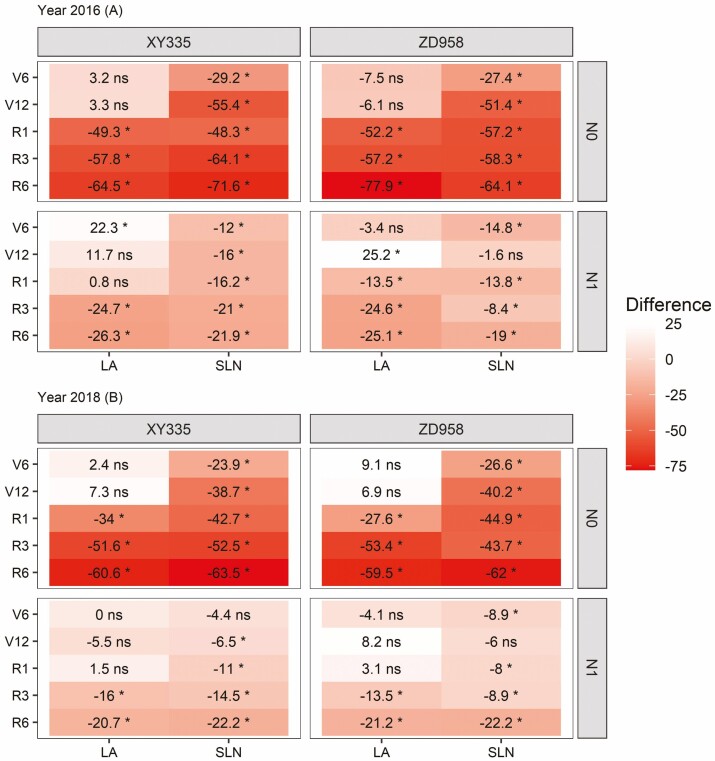
Heatmap of the differences between N applications N0 and N1 compared with N2 application for XY335 and ZD958. Canopy SLN (g m^−2^); leaf area per plant (LA, m^2^); *P < 0.05, ns = non-significant.

Green leaf area per plant (LA) and canopy average leaf N content per unit area (SLN) differed between the N application rate, stages and between both hybrids. In most cases, the effects of the three factors depended on each other **[see****Supporting Information—Table S1****]**. Leaf area reached a maximum value at the R1 stage and then decreased as leaves senesced. There was no difference in LA among N treatments during vegetative stages. However, from the R1 stage LA values in N0 were lower than in N1 and N2, and in N1 were lower than in N2 from the R3 stage (Figs 1A and 2). Compared with the N2 treatment, the LA difference increased with growth stages in N0 and N1 treatments. For N0 this difference increased to 60–78 % at the R6 stage, and for the N1 this difference became 19–26 % (Fig. 2). Canopy SLN decreased gradually during growth stages in N0. However, at the other two higher N levels, SLN increased up to the V12 stage and then decreased during reproductive stages (Fig. 1B). Thus, SLN at the N0 level was consistently lower than SLN at the other two N levels, and the difference between N0 and N2 increased from 20–30 % at the V6 stage to 60–70 % at the R6 stage. In N1 this difference increased from 12–15 % at the V6 stage to 19–23 % at the R6 stage (Fig. 2). These differences in overall responses to N treatments were associated with differences in initial soil N (Table 2). Therefore, in response to low N, the maize plants tended to maintain or sometimes even slightly increase LA and decrease the SLN during vegetative stages to adapt to N deficiency. However, both LA and SLN decreased at reproductive stages, and the proportional reduction was similar for both traits (Fig. 2).

### LB and total leaf N content variations

**Figure 3.**
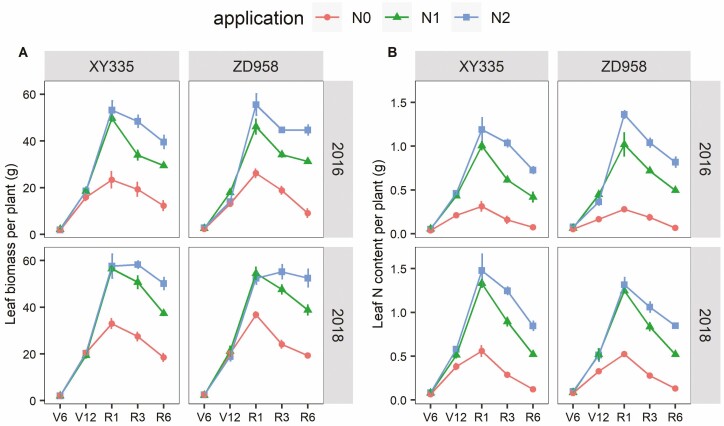
Leaf biomass per plant (A) and the leaf N content per plant (B) at five growth stages among three N application rates of XY335 and ZD958 during the 2016 and 2018 growing seasons.

At different N levels, maize plants had similar LB during vegetative stages, except for the V12 stage of ZD958 in 2016 (Fig. 3A). The LA in N1 was higher than the other two N levels leading to this exception (Fig. 1A). During the reproductive stages, there was an increase in LB with increasing levels of N supply. As of the R1 stage, leaves in N0 had significantly lower biomass than N1 and N2, and the LB difference between N1 and N2 mainly started from the R3 stage (Fig. 3A). The total leaf N content per plant (TLN) increased from V6 to R1 stage and decreased thereafter. However, the TLN difference between N0 and other N treatments was significant throughout the whole growth cycle, and the TLN difference increased further with the growth stages (Fig. 3B). The TLN difference between N1 and N2 started at the R3 stage, except for ZD958 in 2016.

### The scaling relationship between LA and LB

**Figure 4.**
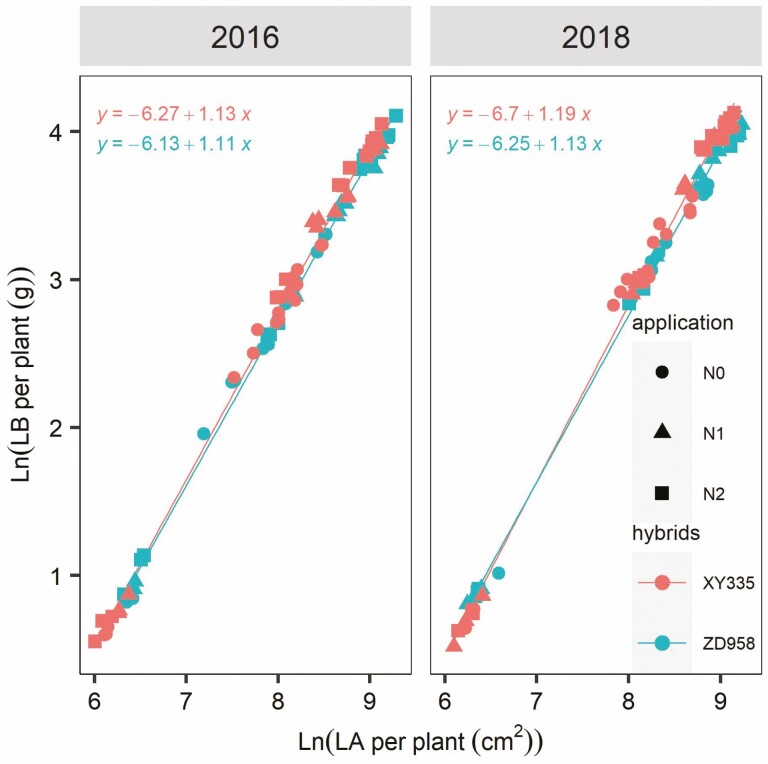
Relationship of leaf biomass (LB, g) and leaf area (LA, cm^2^) per plant of XY335 and ZD958 under three N treatments in 2016 and 2018. Leaf area and LB were ln-transformed. Statistical fits of the power function ln(LB) = ln(*α*) + *β* ln(LA). *α* and *β* are fitted parameters.

For both ZD958 and XY335, *β* values in Equation (5) were greater than 1 in both years, which suggested that LB tended to increase disproportionally faster than LA (Fig. 4). Consequently, canopy SLA decreased with increasing leaf size at the plant level, because SLA was defined as the ratio between LA and LB. The *β* mean values of XY335 were higher than that of ZD958 in 2016 (1.13 vs. 1.11) and 2018 (1.19 vs. 1.13), which implied that in XY335 SLA declined more strongly with LA than in ZD958, suggesting XY335 invested more photosynthetic products into leaf structure building (Fig. 4). When correcting for differences in plant size, the N application rate did not affect the scaling relationship between LA and LB, given the whole growth stages (Table 3).

### The dynamic characteristics of SLA within maize canopy

**Figure 5.**
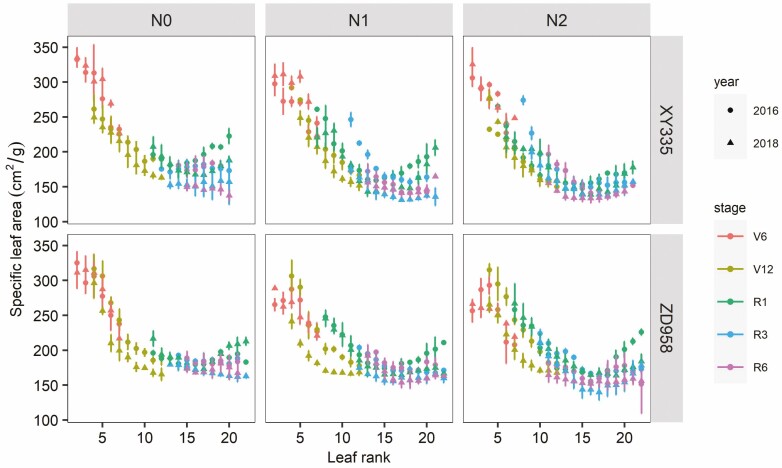
Relationship between SLA (cm^2^ g^−1^) and leaf rank among three N application rates and five stages of XY335 and ZD958 in 2016 and 2018.

Specific leaf area of individual leaves within maize canopy changed with leaf rank, growth stages, N application and hybrids **[see****Supporting Information—Table S2****]**. Specific leaf area decreased with increasing leaf rank during vegetative stages, indicating an increase in SLA from the top canopy towards the bottom of the canopy (note that leaf ranks were counted from the bottom canopy to the top canopy). During reproductive stages, SLA decreased with leaf rank and then slightly increased in the top canopy (Fig. 5).

### Canopy SLA response to N deficiency

**Figure 6.**
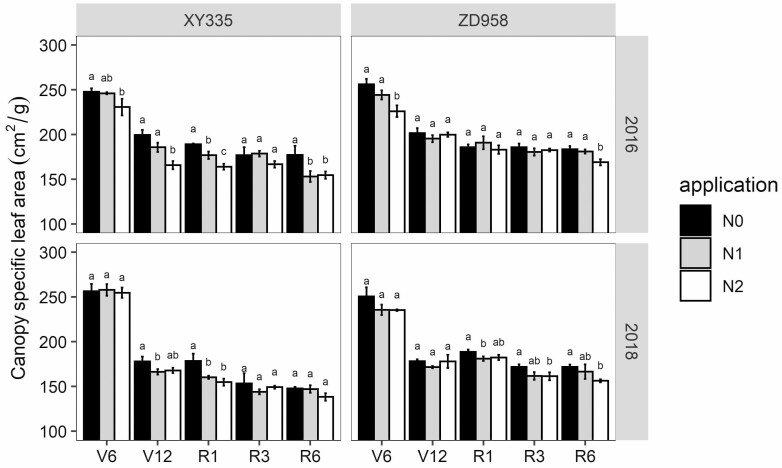
Canopy SLA (cm^2^ g^−1^) at five growth stages among three N application rates of XY335 and ZD958 during the 2016 and 2018 growing seasons. Different lowercase letters indicate significant differences between treatments at *P* < 0.05. The vertical dot lines indicate the shift from vegetative to reproductive growth.

Hybrids, N application rate, growth stages and their interactions had a significant effect on canopy SLA **[see****Supporting Information—Table S2****]**. Canopy SLA sharply declined during vegetative stages, and declined further but less steeply during the reproductive stage (Fig. 6), and this was associated with the vertical SLA distribution within the maize canopy (Fig. 5). Leaves with lower ranks in the part of the lower canopy had higher SLA but senesced faster, leading to the sharp decline of canopy SLA during the vegetative stage as it were the lower-canopy higher-SLA leaves that were dropped. The SLA of the middle and upper leaves in the canopy were relatively stable; hence, the canopy SLA decreased slightly over time during reproductive stages (Fig. 6; **see****Supporting Information—Fig. S2**).

Among N applications, both individual LA and leaf lifespan were significantly smaller under N0 treatment than in the other two N treatments in both hybrids **[see****Supporting Information—Figs S1****and****S2****]**. The two hybrids tended to increase SLA in response to N deficiency regardless of the growth stages. In 2016 for XY335, SLA in the N0 treatment was higher than in the other treatments but this was not the case in ZD958 as indicated by the significant difference between hybrids and N application interaction in 2016. In 2018 there was a trend towards higher SLA values in both hybrids. In 2018 the decline in SLA with growth stage was larger in XY335 than in ZD958 as indicated by the interaction effect between hybrids and stage in that year **[see****Supporting Information—Table S2****]**.

## Discussion

### Trade-off between LA and SLN in response to N deficiency

At the canopy level, crops can respond to N deficiency through a reduction in either LA (reducing light capture), SLN (reducing photosynthetic RUE) or both (Lemaire *et al.* 2008). Different response patterns to N deficiency have been associated with different crop species (Lemaire *et al.* 2008), whereby maize was denoted as a species that tends to maintain its LA at the expense of SLN under N deficiency (Vos *et al.* 2005). Previous research mainly focused on the vegetative growth stages (Lemaire *et al.* 2008; Oosterom *et al.* 2010; Massignam *et al.* 2011). Our findings confirm that maize responds to N stress by the maintenance of LA at the expense of SLN during vegetative stages, but extend this by showing that maize reduced both LA and SLN about equally during the reproductive stages (Fig. 2). This suggests that part of the photosynthetic assimilates and N stored in the vegetative organs were translocated and used for grain filling during reproductive stage (Wei *et al.* 2019; Liu *et al.* 2022). In general, increasing N supply to the crop might improve LA, prolong leaf lifespan and boost photosynthesis (Antonietta *et al.* 2019; Li *et al.* 2022); however, in our study, there was no remarkable difference in LA across N treatments throughout the vegetative stages (Fig. 1). This was primarily caused by the SLA of the N0 treatment being higher than that of other N-fertilized treatments (Figs 1 and 2). Probably the higher-SLA leaves could capture more light by spreading a given amount of leaf N over a greater area and allocating a greater fraction to photosynthesis, than by overlapping it in a given area (Knops and Reinhart 2000). As a result, maize plants tended to increase SLA to maintain LA when N was scarce.

### Leaf morphological plasticity in response to N deficiency

Individual LA and leaf lifespan within plant canopies determine the temporal and spatial distribution of green leaf area and the ability of the crop to intercept light at the canopy level (Simioni *et al.* 2004; Perez *et al.* 2019). Nitrogen supply affects both LA development and leaf senescence (as shown in **Supporting Information—Figs S1****and****S2**), and consequently the LA distribution in the canopy (Vos *et al.* 2005; Bonelli and Andrade 2020). However, the negative effect of N deficiency on individual LA was apparent only in the middle to upper leaf ranks, i.e. upwards of leaf rank 10–12 **[see****Supporting Information—Fig. S1****]**. This explains why the canopy LA was not affected by N deficiency at the V6 and V12 stages, and the results agree with previous studies under field conditions (Dwyer *et al.* 1992; Fan *et al.* 2020). Smaller upper leaves allow more light to penetrate the maize canopy, which improves photosynthesis of middle and lower leaves and enhances RUE, as photosynthesis of leaves lower in the canopy is often strongly light-limited (Boomsma *et al.* 2009; Kant *et al.* 2011). Such an increase in RUE could thus be a positive effect of the upper leaf size reduction under low N found here. Additionally, the onset of leaf senescence begins earlier when N uptake and allocation to leaves is insufficient, and senescence of leaves below the ear leaf (leaf rank 9–12) was most sensitive to N availability **[see****Supporting Information—Fig. S2****]**. Accelerating the senescence of lower leaves allows more resources (e.g. nitrogen and carbohydrates) to be reinvested in the production of younger leaves and later on in reproduction, and such remobilization and translocation become more important at low N (Massignam *et al.* 2011).

### The relationship between LA and LB

Specific leaf area (cm^2^ g^–1^) is an essential functional trait since it indicates the amount of light-capturing surface area that leaves have invested per unit of dry mass (Milla and Reich 2007; Pérez-Ramos *et al.* 2013; Liu *et al.* 2017). Leaf biomass scaled disproportionately faster than LA, as indicated by the scaling exponents *β* > 1 in Equation (5) in XY335 and ZD958 (Fig. 4). However, the scaling exponent *β* of XY335 was somewhat larger than that of ZD958, indicating that for a given canopy size, XY335 tended to have lower SLA and that LB typically increased faster with increasing LA in XY335 than in ZD958 (Fig. 4). These findings suggest that the biomass costs (associated with developing and maintaining leaf structure) of deploying light-absorbing LA are greater for XY335 than ZD958 (Chen *et al.* 2014; Li *et al.* 2019). Lower SLA (thicker leaves) is typically correlated with longer or more palisade cells and larger bundle-sheath cells. This provides space for more or larger chloroplasts and hence a greater amount of photosynthetic enzymes, increasing photosynthetic capacity per unit of LA (Evans and Poorter 2001; Mu *et al.* 2016; Yao *et al.* 2016). This is supported by the finding that XY335 has a greater net photosynthetic rate and higher photosynthetic N-use efficiency compared to ZD958 (Chen *et al.* 2014, 2016). ZD958 displayed stay-green characteristics throughout the grain-filling stage, due to higher leaf number and lower leaf N remobilization efficiency in comparison to XY335 (Chen *et al.* 2014; Liu *et al.* 2021). Intriguingly, there appears to be no difference in yield potential between these two hybrids, suggesting that positive effects of the stay-green behaviour of ZD958 compensated for its lower photosynthetic capacity (Li *et al.* 2022). The connection between LA and LB at the plant level was not substantially different between N treatments after accounting for the impact of canopy size (Table 3). This is most likely because N treatments had minimal impact on LA during vegetative phases due to the maize adaptation strategy, and the LB was driven by LA build-up (Vos *et al.* 2005; Oosterom *et al.* 2010).

### Spatial and temporal distribution of SLA within maize canopy

**Figure 7.**
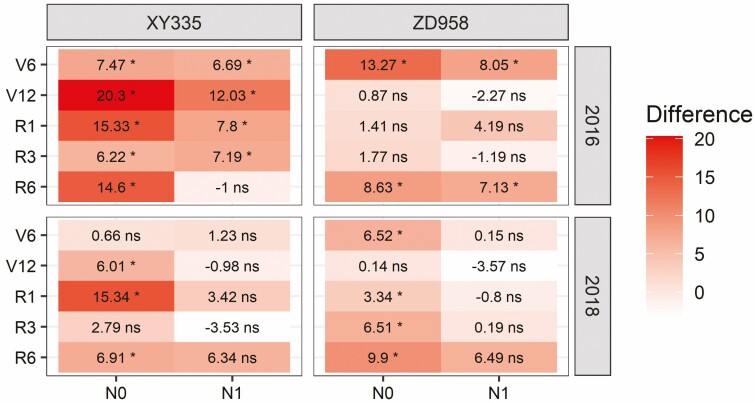
Heatmap of the differences in canopy SLA (cm^2^ g^−1^) between N applications compared with N2 application of XY335 and ZD958 in 2016 and 2018. Significance level: *P < 0.05, ns = non-significant.

Capturing spatial and temporal variability in SLA is crucial in crop growth simulation models. However, many modelling studies use a fixed SLA value even though SLA varies as a function of leaf size, leaf age, growth rate and climate conditions (Yao *et al.* 2016; Liu *et al.* 2017; Serbin *et al.* 2019; Zhou *et al.* 2020). Specific leaf area within the maize canopy varied with leaf rank (Fig. 5), leaves in the lower canopy had larger SLA, while the middle and upper leaves’ SLA stayed the same or even increased somewhat with leaf rank, indicating that they may be thicker than lower leaves (Knops and Reinhart 2000; Yao *et al.* 2016). This higher SLA of lower leaves allows more light to be captured in the lower part of the canopy, and as leaves there are light-limited, possible reductions in SLN and associated photosynthetic capacity will not have a large negative effect (Knops and Reinhart 2000). We observed that canopy SLA significantly decreased during the vegetative stages and slowly decreased during the reproductive stages across all N treatments (Fig. 6). However, the N0 treatment had a slightly higher canopy SLA during the vegetative stage (Fig. 7). This probably because leaves in the lower part of the canopy have a shorter leaf lifespan **[see****Supporting Information—Fig. S2****]** and higher SLA (Serbin *et al.* 2019; Zhou *et al.* 2020). Faster leaf senescence in N0 plants should negatively affect canopy SLA as it is the lower-canopy leaves that were dropped **[see****Supporting Information—Fig. S2****]**, which generally had relatively high SLA values (Fig. 5). These two contrasting leaf sizes and senescence effects may explain why canopy SLA in the reproductive phase no longer differed between N treatments.

## Conclusion

The current study confirmed and extended the trade-off in maize plants’ response to N deficiency in terms of LA and SLN. At low N availability, maize tended to maintain LA and decrease SLN during vegetative stages, while both LA and SLN declined comparably during the reproductive stage. Individual leaf size, lifespan and SLA within maize canopy contribute to shaping canopy SLA. Canopy SLA decreased with time mainly because leaves that senesce are lower-canopy leaves that have higher SLA than upper leaves that remain. Maize plants tend to increase SLA to adapt to N-deficient conditions, and this could partly offset the negative effects of N deficiency.

## Supporting Information

The following additional information is available in the online version of this article—

Table S1. ANOVA analysis of leaf area per plant (LA) and canopy-averaged SLN of XY335 and ZD958 under different N treatments during the two experimental years.

Table S2. Analysis of variance results for canopy SLA. 

Figure S1. Individual leaf area (cm^2^) of fully expanded leaves versus leaf rank among three N application rates of XY335 and ZD958.

Figure S2. Leaf lifespan (days) of individual leaf rank among three N application rates of XY335 and ZD958.

plac053_suppl_Supplementary_Materials

## Data Availability

The data underlying this article are available in the article and in its online **Supporting Information**.

## Notes

**Plants, Ecosystems & Climate. Chief Editor:** Mary Heskel
