# Supplementary material for: Plastic response of leaf traits to N deficiency in field-grown maize
Source: AoB Plants. 2022 Oct 27;14(6):plac053. doi: 10.1093/aobpla/plac053 (PMC9762715; doi:10.1093/aobpla/plac053)
Supplement: plac053_suppl_Supplementary_Materials [file plac053_suppl_supplementary_materials.docx]

**Supporting information**

**Table S1**. ANOVA analysis of leaf area per plant (LA) and canopy-averaged SLN of XY335 and ZD958 under different N treatments during the two experimental years.

|  | Year 2016 | | Year 2018 | |
| --- | --- | --- | --- | --- |
|  | LA | SLN | LA | SLN |
| Hybrids (H) | *** | ns | *** | *** |
| N application (N) | *** | *** | *** | *** |
| Stage (S) | *** | *** | *** | *** |
| H×N | *** | ** | ns | ** |
| H×S | *** | ** | *** | *** |
| N×S | *** | *** | *** | *** |
| H×N×S | *** | ** | ns | * |

LA = leaf area per plant (m^2^); SLN = canopy-averaged leaf N content per unit leaf area (g/m^2^). Significance level: * P < 0.05, ** P < 0.01, *** P < 0.001, ns = non-significant.

**Table S2.** Analysis of variance results for canopy SLA

| Variance | Canopy SLA | |
| --- | --- | --- |
|  | Year 2016 | Year 2018 |
| Hybrids (H) | *** | *** |
| N application (N) | *** | *** |
| Stage (S) | *** | *** |
| H×N | *** | ns |
| H×S | ns | *** |
| N×S | *** | ** |
| H×N×S | ** | * |

Significance level: * *P* < 0.05, ** *P* < 0.01, *** *P* < 0.001, ns = non-significant.


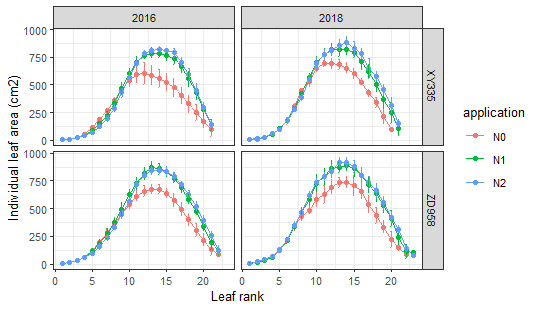


**Figure. S1** Individual leaf area (cm^2^) of fully expanded leaves versus leaf rank among three N application rates of XY335 and ZD958. The dataset of individual leaf areas in 2018 was published and cited by *Li et al.* (2022).


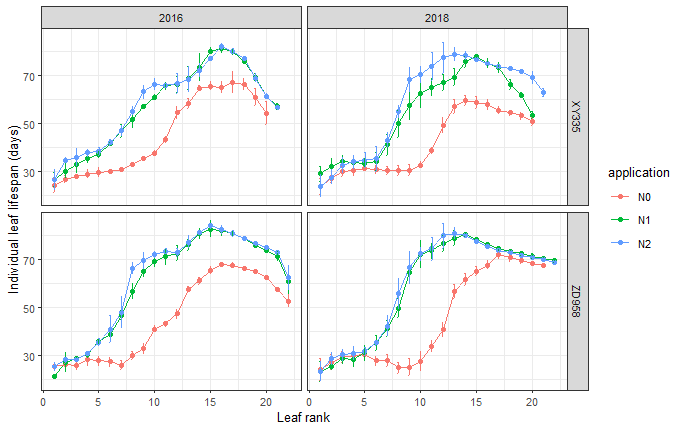


**Figure. S2** Leaf lifespan (days) of individual leaf rank among three N application rates of XY335 and ZD958. The dataset of individual leaf lifespans in 2018 was published and cited by *Li et al.* (2022).
